# Supplementary material for: A cellular trafficking signal in the SIV envelope protein cytoplasmic domain is strongly selected for in pathogenic infection
Source: PLoS Pathog. 2022 Jun 17;18(6):e1010507. doi: 10.1371/journal.ppat.1010507 (PMC9275724; doi:10.1371/journal.ppat.1010507)
Supplement: S11 Fig — SGS of plasma virus from the indicated time points is shown for 4 animals inoculated with SIVmac239ΔGY containing R722G and 3 point mutations (T735I, Q739R, and P744L) shown to confer a new basolateral sorting signal (Fig 8). Amino acid sequences are shown for the Env distal membrane spanning domain and the entire cytoplasmic tail. Amplicons are shown relative to parental SIVmac239 with a.a. identity indicated by a period and deletions indicated by a dash. Plasma viral loads over time for each animal are shown. (PDF) [file ppat.1010507.s011.pdf]

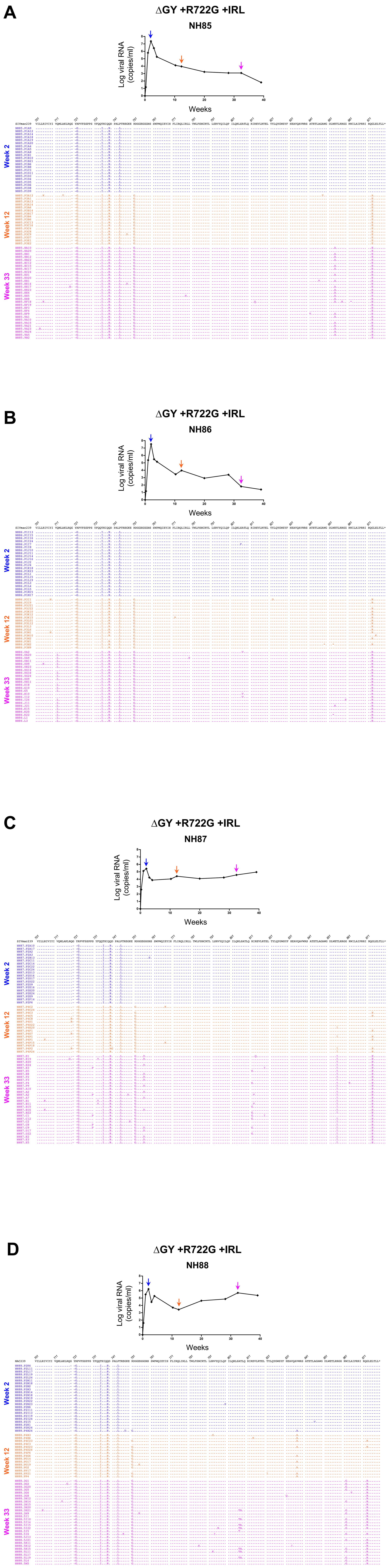
A
ΔGY +R722G +IRL
NH85
Log viral RNA (copies/ml)
Weeks
Week 2
Week 12
Week 33
B
ΔGY +R722G +IRL
NH86
Log viral RNA (copies/ml)
Weeks
Week 2
Week 12
Week 33
C
ΔGY +R722G +IRL
NH87
Log viral RNA (copies/ml)
Weeks
Week 2
Week 12
Week 33
D
ΔGY +R722G +IRL
NH88
Log viral RNA (copies/ml)
Weeks
Week 2
Week 12
Week 33
