# Supplementary material for: A cellular trafficking signal in the SIV envelope protein cytoplasmic domain is strongly selected for in pathogenic infection
Source: PLoS Pathog. 2022 Jun 17;18(6):e1010507. doi: 10.1371/journal.ppat.1010507 (PMC9275724; doi:10.1371/journal.ppat.1010507)
Supplement: S7 Fig — SGA sequencing of plasma virus from the indicated time points is shown for animals inoculated with SIVmac239ΔGY containing both R722G and ΔQTH mutations (Panels A, B and C), or ΔGY containing R722G (Panels D, E and F). Amino acid sequences (a.a. 701–879) are shown for the Env distal membrane spanning domain and the entire cytoplasmic tail. Amplicons are shown relative to parental SIVmac239 with a.a. identity indicated by a period and deletion mutations indicated by a dash. Plasma viral loads over time for each animal are shown. (PDF) [file ppat.1010507.s007.pdf]

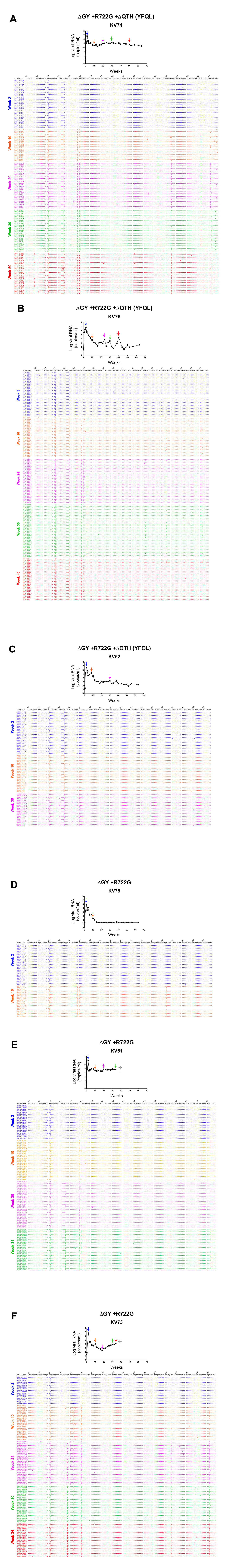
A
ΔGY +R722G +ΔQTH (YFQL)
KV74
Log viral RNA (copies/ml)
Weeks
Week 2
Week 10
Week 20
Week 30
Week 50
B
ΔGY +R722G +ΔQTH (YFQL)
KV76
Log viral RNA (copies/ml)
Weeks
Week 3
Week 10
Week 24
Week 30
Week 40
C
ΔGY +R722G +ΔQTH (YFQL)
KV52
Log viral RNA (copies/ml)
Weeks
Week 2
Week 10
Week 30
D
ΔGY +R722G
KV75
Log viral RNA (copies/ml)
Weeks
Week 2
Week 10
E
ΔGY +R722G
KV51
Log viral RNA (copies/ml)
Weeks
Week 2
Week 10
Week 20
Week 34
F
ΔGY +R722G
KV73
Log viral RNA (copies/ml)
Weeks
Week 2
Week 10
Week 24
Week 30
Week 34
