# Supplementary material for: A cellular trafficking signal in the SIV envelope protein cytoplasmic domain is strongly selected for in pathogenic infection
Source: PLoS Pathog. 2022 Jun 17;18(6):e1010507. doi: 10.1371/journal.ppat.1010507 (PMC9275724; doi:10.1371/journal.ppat.1010507)
Supplement: Attachment — Submitted filename: Response to reviewers.docx [file ppat.1010507.s017.docx]

The Editors

PLOS Pathogens

March 10th 2022

Dear Editors,

Herewith, we are submitting a revised version of our paper entitled "A cellular trafficking signal in the SIV envelope protein cytoplasmic domain is strongly selected for in pathogenic infection". We were extremely pleased to read the very positive comments of the three reviewers of our original submission, and their views that this paper describes important new insights to the biology of primate lentiviruses that expand our understanding of the mechanisms underlying pathogenesis as well as potential application in vaccine development.

We have considered the comments of the two reviewers who raised minor, but nevertheless important, concerns and we have addressed these as described in the ‘responses to reviewers.

We attach two versions of the modified manuscript; one in which our changes have been highlighted in the ‘track changes’ mode and one ‘clean’ version.

We hope that these modifications are acceptable to yourselves and the reviewers and that the paper can now be published in PLOS Pathogens.

Yours sincerely,

Mark Marsh and James Hoxie

Co-corresponding authors

Responses to reviewers #1 and #3

Reviewer #1:

Minor points are the use of historical controls, and the question of whether SIVmac239 (a widely accepted model virus for HIV pathogenesis) is completely reflective of other SIV/HIV viruses. However, these are minor points that do not detract from this overall laudable study.

We thank the reviewer for his/her very positive comments on the paper. With regard to the use of historical controls, we have accumulated a considerable amount of data on SIVmac239 infection in pig tail macaques (PTM) over many years. The results from multiple animals are highly reproducible and similar to those obtained by others. It is now accepted practice to use these historical data rather than additional control animals in experiments with primates, both to reduce the number of animals used and the expenses involved.

With respect to SIVmac239, it is clear this virus is not HIV. Nevertheless, SIVmac239 infection of PTM is now a well-accepted model for HIV infection in humans. Moreover, as we noted, the trafficking signals we describe in the Env cytoplasmic for SIV are highly conserved in all HIVs and have been well characterized for both viruses *in vitro* for both endocytosis and polarized sorting. Our introduction cites many of the references for HIV-1 Env that make this point. Given the conservation of these motifs, both genetically and functionally, the many parallels of nonhuman primate models of SIV infection with AIDS in humans, we are of the view that the ∆GY model and the new data we present provides information about host-virus interaction that is relevant to HIV infection in man.

Reviewer #3:

1. Figure 2 shows a very reasonable measurement of cell surface and virion Env for the ΔGY and revertant viruses by surface biotinylation, pull down, and immunoblotting. Flow cytometry data establishing levels of cell surface Env would add to this as a direct quantification of cell surface Env levels for deltaGY and the revertant viruses.

2. Figure 3B demonstrates subcellular localization by immunofluorescence microscopy. While some differences in subcellular localization can be appreciated as shown, the use of maximum projections does not allow the conclusion that the diffuse signal seen with deltaGY, deltaGY +R722G, deltaGY +S727P is on the cell surface. A supplemental figure with a confocal section at the coverslip level vs. mid-cell level would better establish this PM distribution.

Again, we thank the reviewer for his very positive comments on the paper. In response to point 1, we have done flow cytometric analysis of cell surface Env expression and found this closely replicates the biochemical data generated using the biotinylation assay. However, we have not used flow cytometry routinely, because we found the data from biotinylation experiments and pull-downs to be more useful as it allowed us to monitor both total cell-associated Env and cell surface Env in the same samples. In this way we could exclude changes in total cellular Env expression as the basis for changes in cell surface expression. Below we include a representative image for the reviewer in which we show data from BC7 cells using both flow cytometry and the biotinylation assay. We see the same general trend in cell surface Env expression using both assays, i.e., cell surface Env decreases with ∆GY, that this is rescued with the R722G and S727P substitutions. R722G + ∆QTH give near wt levels of cell surface Env. As these results are from a single experiment, we do not consider them appropriate for inclusion in the manuscript.

Figure legend: **Quantification of cell surface Env in BC7 cells**.

A CD4/CCR5 negative human T-cell line (BC7) was infected with VSV-G pseudotyped SIVmac239 for 24 hr and the expression of cell surface SIVmac239 Env or Envs containing the indicated substitutions was determined by biotinylation, pull-down and western blot or flow cytometry. Results are expressed as the signal from Env (intensity of bands on western blots or MFI from flow cytometry) normalized to intensity of bands or MFI of cellular Gag p57/p27 and as a value relative to SIVmac239 Env set to 1.

2. We accept the reviewer’s comment regarding the cellular distribution of the various constructs illustrated by projections. We have added a supplemental figure (Fig. S2C) showing optical sections through the top, middle and bottom (adjacent to the coverslip) of cells expressing each of the constructs illustrated in Figure 3B and 3C. Fig S2C clearly shows ∆GY, ∆GY +R722G, ∆GY +S727P on the top and bottom surfaces (as well as some inside the cell which is presumably within the exocytic and endocytic pathways). By contrast, SIVmac239, ∆GY +∆QTH (YFQL) and ∆GY +R722G+∆QTH (YFQL) are only observed inside the cells. The text on page 10 and figure legend S2 have been altered accordingly.
